# Bergamottin Suppresses Metastasis of Lung Cancer Cells through Abrogation of Diverse Oncogenic Signaling Cascades and Epithelial-to-Mesenchymal Transition

**DOI:** 10.3390/molecules23071601

**Published:** 2018-07-02

**Authors:** Jeong-Hyeon Ko, Dongwoo Nam, Jae-Young Um, Sang Hoon Jung, Gautam Sethi, Kwang Seok Ahn

**Affiliations:** 1 Department of Science in Korean Medicine, College of Korean Medicine, Kyung Hee University, Seoul 02447, Korea; gokjh1647@gmail.com (J.-H.K.); hanisanam@hanmail.net (D.N.); jyum@khu.ac.kr (J.-Y.U.); 2 KHU-KIST Department of Converging Science and Technology, Kyung Hee University, Seoul 02447, Korea; shjung507@gmail.com; 3 Department for Management of Science and Technology Development, Ton Duc Thang University, Ho Chi Minh City 700000, Vietnam; 4 Faculty of Pharmacy, Ton Duc Thang University, Ho Chi Minh City 700000, Vietnam; 5 Department of Pharmacology, Yong Loo Lin School of Medicine, National University of Singapore, Singapore 117600, Singapore

**Keywords:** bergamottin, EMT, metastasis, lung cancer

## Abstract

Bergamottin (BGM) is a naturally occurring furanocoumarin and is known to inhibit the growth of tumor cells. However, there is no available evidence that BGM has an inhibitory effect on cancer metastasis, specifically on the epithelial-to-mesenchymal transition (EMT) process in the malignant cells. Here we aimed to evaluate the antimetastatic potential of BGM in human lung adenocarcinoma cells. Our results demonstrate that BGM can block EMT, and observed inhibition was accompanied by downregulation of fibronectin, vimentin, N-cadherin, twist and snail expression, and upregulation of occludin and E-cadherin. Interestingly, transforming growth factor-β (TGF-β)-induced upregulation of fibronectin, vimentin, N-cadherin, twist and snail, and downregulation of occludin and E-cadherin, were abrogated by BGM treatment. Moreover, the treatment of BGM repressed TGF-β-induced cell invasive potential. BGM treatment also inhibited multiple oncogenic cascades such as PI3K/Akt/mTOR. Overall, the results demonstrate the potential antimetastatic activity of BGM against lung cancer cells.

## 1. Introduction

The epithelial-to-mesenchymal transition (EMT) is a reversible process where epithelial cells convert to mesenchymal cells with migratory and invasive properties, and it has been implicated in several pathological conditions. Recently, EMT has also been reported to increase the aggressiveness of tumor cells, contributing to their increased migration, invasion and metastasis [1,2,3,4]. EMT can be caused by several growth factors, including transforming growth factor-β (TGF-β), epidermal growth factor (EGF), fibroblast growth factor (FGF) and hepatocyte growth factor (HGF), and Wnt and several other signaling pathways have been implicated in its regulation [4,5,6]. TGF-β-stimulated cells show mesenchymal-like spindle-shaped morphology accompanied by loss in cell–cell adhesion and decrease of polarity [3,7,8]. Targeting of the TGF-β-induced EMT and the signaling pathway could have broad biological significance and potentially guide the development of a novel intervention against cancer metastasis.

Metastasis is a critical hallmark of cancer cells and is a complicated process, where cells spread from their site of origin to distant organs [3,4,9,10,11,12]. To carry out the metastatic process, cancer cells can often detach from the primary tumor site, extravasate or invade, and grow at distant organs [13,14,15]. It is estimated that metastasis is responsible for around 90% of mortality in cancer patients [10,16], and several lung cancer patients are diagnosed with metastatic disease. These facts highlight the need for further understanding of molecular mechanisms of metastasis and to develop new therapeutic approaches to target this process.

The need for the development of novel anticancer drugs with lower toxicity has led to reports analyzing the potential of useful anticancer compounds in fruits, vegetables and herbs [17,18,19,20,21,22]. Bergamottin (BGM) is a major furanocoumarin found in grapefruits (*Citrus paradise*). Grapefruit furanocoumarins can display several pharmacological effects including anticancer activities as well as bone health-promoting effects [23]. BGM has shown to be a potential anticancer agent in various tumor cell lines and preclinical mouse models [24,25,26,27]. We have previously demonstrated that BGM inhibited multiple myeloma cell growth and caused programmed cell death through the abrogation of the STAT3 signaling pathway [26]. Furthermore, BGM when used in combination with simvastatin was found to exert enhanced anticancer effects against human chronic myelogenous leukaemia via abrogation of the NF-κB abrogation pathway [28]. Hwang et al. [25] have reported the antitumor effects of BGM in abrogating invasion of human fibrosarcoma cells as well as on phorbol-12-myristate-13-acetate-promoted MMP-9 activity. Here we reveal for the first time that BGM is able to efficiently inhibit EMT in lung cancer cells. Our current study conclusively suggests that BGM might function as an efficient modulator of EMT and metastasis.

## 2. Materials and Methods

### 2.1. Reagents

Bergamottin (BGM) was obtained from Santa Cruz Biotechnology (Santa Cruz, CA, USA). Recombinant human TGF-β was purchase from Millipore (Bedford, MA, USA). 3-(4,5-Dimethylthiazol-2-yl)-2,5-diphenyltetrazolium bromide (MTT) and 4,6-diamidino-2-phenylindole (DAPI) were from Sigma-Aldrich (St. Louis, MO, USA). RPMI 1640, fetal bovine serum (FBS), and antibiotic–antimycotic mixture were obtained from Thermo Fisher Scientific Inc. (Waltham, MA, USA).

### 2.2. Cell Culture and Treatments

Human lung carcinoma A549 and H1299 cells were obtained from the American Type Culture Collection (Manassas, VA, USA). Cells were grown in RPMI 1640 medium supplemented with 10% FBS, penicillin (100 units/mL), and streptomycin (100 μg/mL), and maintained at 37 °C under 5% CO_2_ atmosphere. Cells were treated by BGM directly to medium to give the final concentrations (30–100 μM) for described time periods.

### 2.3. MTT Assay

Cells were seeded in 96-well plates at a density of 1 × 10^4^ cells/well. Thereafter, cells were treated with various BGM doses and MTT assay was performed as described previously [8].

### 2.4. Western Blot Analysis

Whole cell lysates were prepared in the presence of protease inhibitors and resolved in a 10–12% SDS–polyacrylamide gel. Thereafter, Western blot was carried out as described previously [29].

### 2.5. Reverse Transcription Polymerase Chain Reaction (RT–PCR)

Total RNA was extracted with Trizol reagent and was converted to cDNA. RT–PCR was performed using a method reported previously [30].

### 2.6. Immunocytochemistry

Cells were seeded in eight-well glass chamber slides, fixed with cold methanol and permeabilized with 0.2% Triton X-100 in PBS for 15 min, and immunocytochemistry was carried out as described previously [8].

### 2.7. Cell Adhesion Assay

Ninety-six-well plates were coated with 1 μg/mL of gelatin for 1 h at room temperature. The wells were blocked for 30 min at room temperature with 0.5% BSA to avoid nonspecific cell adhesion. Cells were plated at a density of 4 × 10^4^ cells/well in serum-free medium and incubated with TGF-β (10 ng/mL), BGM (100 μM) or the combination. Thereafter, the cells were washed with PBS and attached cells were subjected to MTT assay.

### 2.8. Invasion Assay

Real-time cell analysis (RTCA) of invasion was monitored in the xCELLigence DP instrument (Roche Diagnostics, Mannheim, Germany) as described earlier [31]. Cell index was measured every 15 min for up to 30 h with the RTCA software (version 1.2, Roche Diagnostics).

### 2.9. Boyden Chamber Assay

Invasion assay was performed using a 48-well Boyden chamber (Nuero Probe, Cabin John, MD, USA) as reported previously with minor modifications [32].

### 2.10. Wound Healing Assay

Wound healing assay was done to detect the cell migration ability using established procedure [31].

### 2.11. Statistical Analysis

All experiments were repeated at least two or three times. Data are presented as the mean ± standard deviation (SD) of three experiments. Statistical analysis was carried out using GraphPad Prism version 5 (GraphPad Software, La Jolla, CA, USA) using Student’s *t*-test and ANOVA. Significance was set at *p* < 0.05.

## 3. Results

### 3.1. BGM Inhibits EMT in Lung Cancer Cells

**Figure 1:**
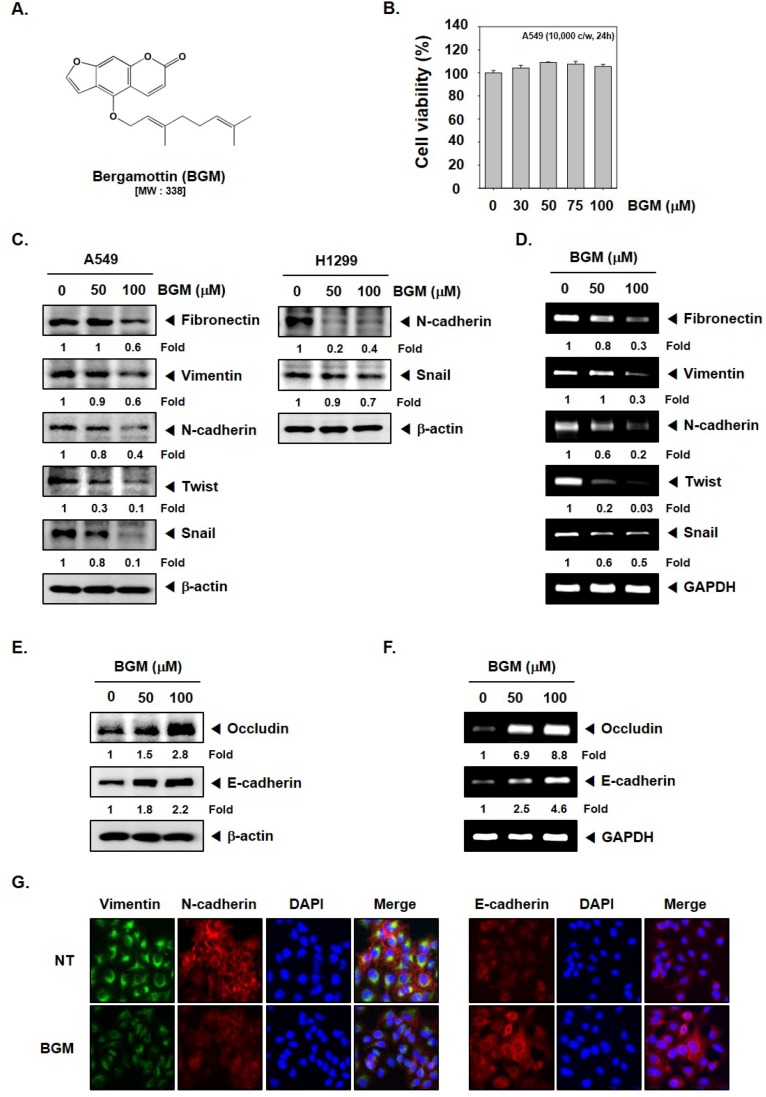
Effects of bergamottin (BGM) on epithelial-to-mesenchymal transition (EMT) in lung cancer cells. (**A**) Chemical structure of BGM. (**B**) A549 cells were treated with BGM (30–100 μM) for 24 h and cell viability was determined by MTT assay. (**C**,**E**) A549 and H1299 cells were treated with BGM and Western blot analysis was performed using various antibodies as indicated above. (**D**,**F**) A549 cells were treated with BGM for 24 h and RT–PCR was performed using different primers as indicated above. Band intensities were estimated by the Image J software (version 1.43u, National Institutes of Health, Bethesda, MD, USA). (**G**) A549 cells were treated with 100 μM BGM for 24 h and immunocytochemistry was performed using three different antibodies as depicted above.

The chemical structure of BGM has been depicted in Figure 1A. We analyzed the cytotoxic effect of BGM in human lung cancer cells using MTT assay. As shown in Figure 1B, exposure to the various concentrations of BGM (0, 30, 50, 75, 100 μM) for 24 h had no significant effect on cell viability. We observed that the expression of pro-EMT markers was downregulated while expression of anti-EMT markers, occludin and E-cadherin, was substantially increased upon the exposure to 50 and 100 μM BGM in lung cancer cells (Figure 1C,E). Additionally, we examined the effect of BGM on EMT marker expression in a different lung cancer cell line, H1299. As shown in Figure 1C right panel, BGM was observed to reduce both the N-cadherin and snail protein levels in H1299 cells. Moreover, the decrease in mRNA expression of these markers upon BGM treatment also correlated with the changes observed in protein levels in A549 cells (Figure 1D,F). We also confirmed expression of various EMT markers by immunocytochemistry and discovered that the expression of pro-EMT markers was downregulated, whereas E-cadherin level was increased in A549 cells (Figure 1G).

### 3.2. BGM Suppresses TGF-β-Induced EMT in Lung Cancer Cells

**Figure 2:**
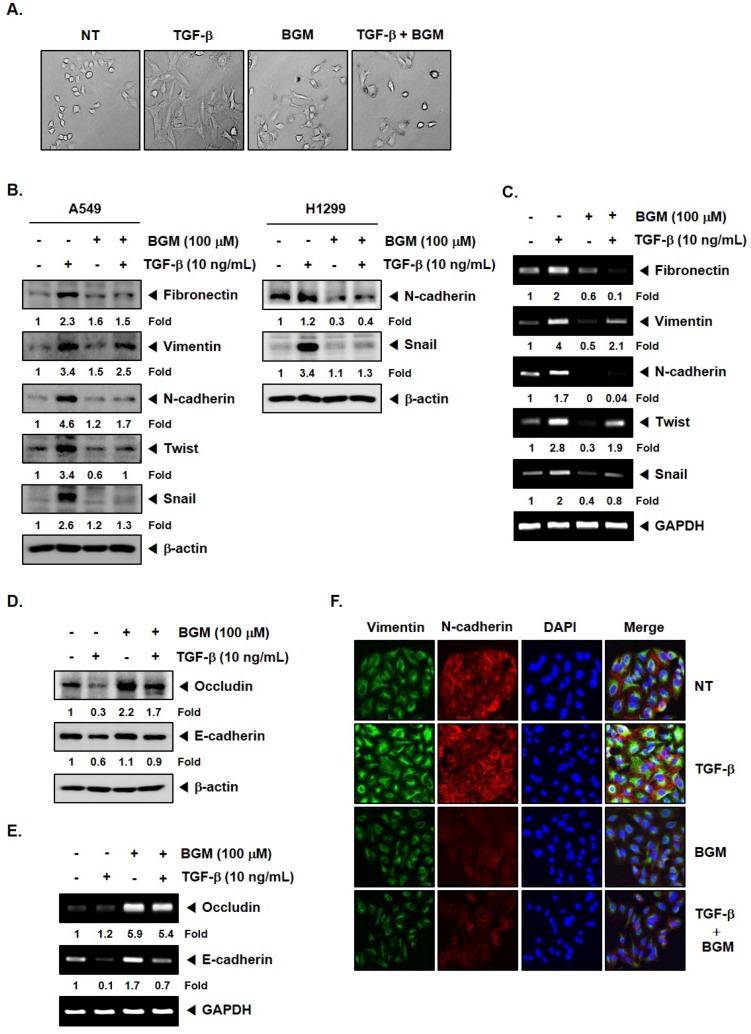
BGM blocks TGF-β-induced EMT process. (**A**) Phase-contrast microscopy for the identification of morphological changes induced in A549 cells upon TGF-β (10 ng/mL), BGM (100 μM), or the combination treatment. (**B**,**D**) Cells were treated as described above in panel A for 24 h and Western blotting was done using various antibodies. (**C**,**E**) A549 cells were treated as described above for 24 h and RT–PCR analysis was carried out using different primers. Band intensities were estimated by the Image J software. (**F**) A549 cells were treated as described above in panel A and immunocytochemistry was performed.

TGF-β can cause and regulate EMT in a variety of cellular models. To determine whether BGM could affect an inducible EMT process, we determined the morphological changes upon treatment with TGF-β, BGM, or the combination group of two (Figure 2A). The exposure to TGF-β substantially caused an elongated and spindle-like morphology. However, BGM exposure substantially reduced TGF-β-induced mesenchymal phenotype (Figure 2A). We also examined the levels of EMT markers upon treatment with TGF-β, BGM, or the combination group of two. As shown in Figure 2B–E, upregulation of anti-EMT protein and mRNA expression and downregulation of occludin and E-cadherin proteins and mRNA expression were effectively prevented upon BGM treatment. Figure 2B right panel revealed that BGM also reduced the expression of TGF-β-induced N-cadherin and snail in H1299 cells. The data was further confirmed by immunocytochemistry, depicting a downmodulation of vimentin and N-cadherin levels upon BGM treatment in TGF-β-stimulated cells (Figure 2F).

### 3.3. BGM Abrogates TGF-β-Induced Metastatic Properties

**Figure 3:**
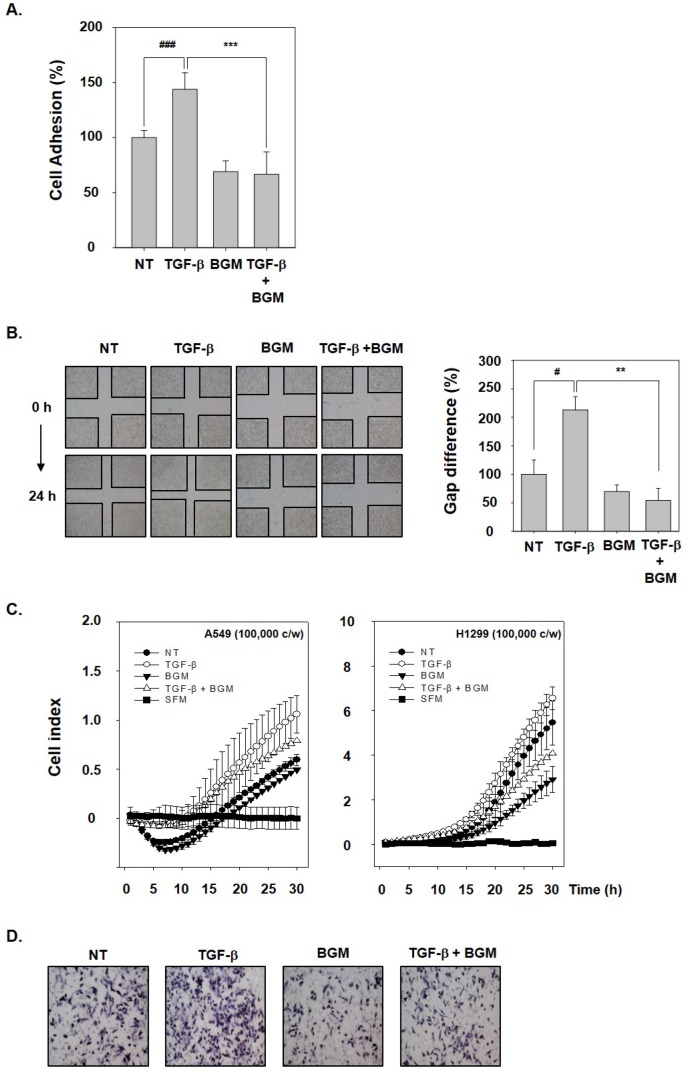
BGM inhibits invasion and migration of lung cancer cells. (**A**) Adhesion of A549 cells to collagen-coated plates with TGF-β (10 ng/mL), BGM (100 μM), or the combination treatment for 40 min. The extent of cell adhesion was quantified by MTT assay. Values are mean ± SD. ^###^ *p* < 0.001 vs. nontreated (NT) cells, *** *p* < 0.001 vs. TGF-β-treated cells. (**B**) To analyze migration, A549 cells were wounded using a sterile tip. The cells were treated as described above to determine the rate of migration. Values are mean ± SD. ^#^ *p* < 0.05 vs. NT cells, ** *p* < 0.01 vs. TGF-β-treated cells. (**C**) Invasion assay was performed with 100,000 cells/well from A549 and H1299 cells in CIM-plate 16 precoated with Matrigel. The rate of invasion was monitored in real-time using the xCELLigence system. (**D**) The anti-invasive effects on A549 cells were detected on the membrane staining with hematoxylin solution.

To analyze the potential consequences of BGM on TGF-β-induced metastatic effects, cells were exposed to TGF-β, BGM, or the combination group of two. It was found that BGM significantly reduced TGF-β-stimulated cell adhesion in A549 cells (Figure 3A). We further investigated the role of BGM in blocking cellular migration using a wound healing assay. TGF-β treatment enhanced wound closure, whereas BGM exposure significantly inhibited the motility and, as a result, the wound remained open (Figure 3B). The effect of BGM on invasion was monitored by xCELLigence CIM-plates configuration. Interestingly, we found that TGF-β promoted cell invasion, whereas BGM attenuated TGF-β-mediated invasion in A549 and H1299 cells (Figure 3C). Moreover, BGM was observed to significantly attenuate TGF-β-induced invasion of A549 cells as demonstrated using a Boyden chamber assay (Figure 3D).

### 3.4. BGM Inhibits TGF-β-Induced PI3K/Akt/mTOR Casacade

**Figure 4:**
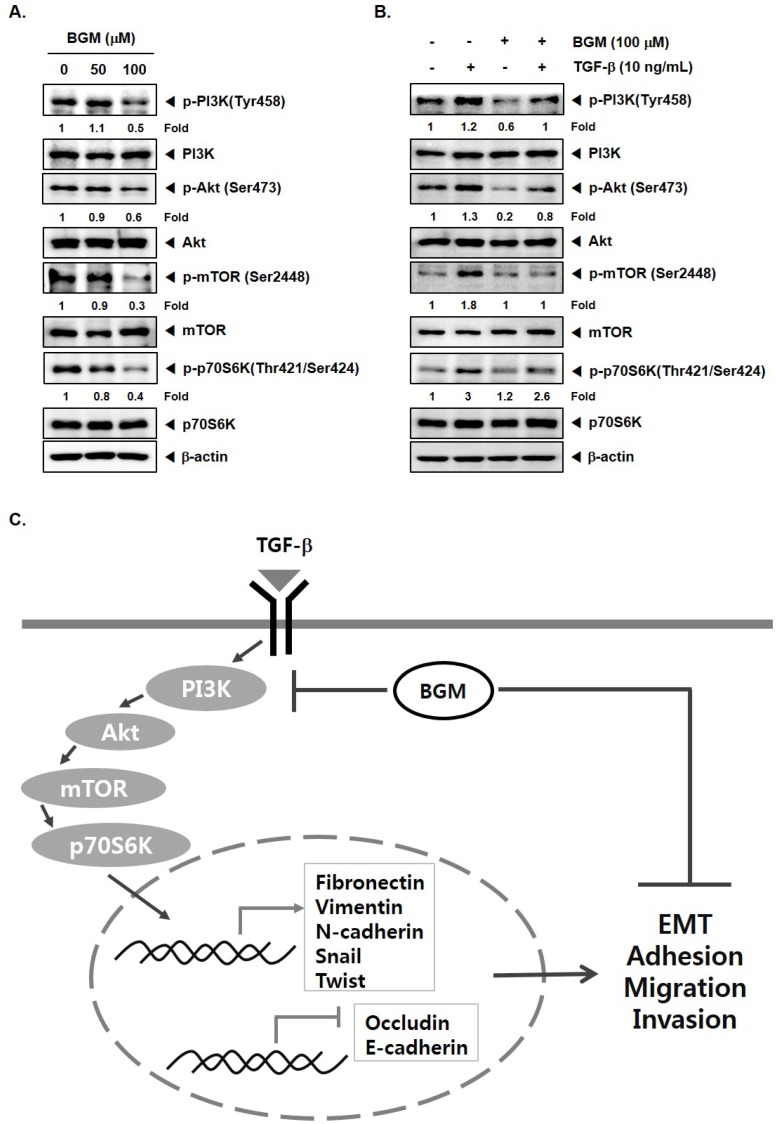
Effects of BGM on Akt activation in TGF-β-treated A549 cells. (**A**) Cells were treated with BGM (50–100 μM) for 6 h and Western blotting was done to detect changes in the phosphorylation status of various kinases as shown above. (**B**) Cells were treated as described above in panel 2A and Western blotting was carried out. Band intensities were estimated by the Image J software. (**C**) A schematic representation of the proposed targets modulated upon BGM treatment.

PI3K/Akt/mTOR kinases have been reported to regulate tumor initiation and metastasis [8,33,34,35,36,37,38]. Previous research has demonstrated that aberrant activation of this oncogenic signal transduction cascade can drive lung cancer metastasis by regulating EMT [39]. Therefore, we next analyzed the role of BGM in modulating PI3K/Akt/mTOR phosphorylation status. As shown in Figure 4A, BGM substantially downregulated the phosphorylation levels of various kinases in A549 cells. Furthermore, it was noted that the treatment of BGM substantially suppressed the TGF-β-mediated increase in activation of these kinases in lung cancer cells (Figure 4B).

## 4. Discussion

Lung cancer remains as one of the primary causes of mortality worldwide. The prognosis of lung cancer remains poor because lung cancer patients rarely display clinical symptoms in earlier stages of the disease. Therefore, rapid exploration of novel intervention targets and therapeutic agents might provide more clinical benefits during lung cancer therapy. Emerging evidence indicates that the EMT process plays an integral part in dissemination of cells into the circulation and can be targeted using pharmacological agents to abrogate the spread of tumor cells effectively. Previous reports have shown a close association between lung cancer progression and EMT [39,40]. Natural compounds have the capability to inhibit survival and metastatic spread of cancer cells [17,18,19,20,21,22,41]. BGM, a bioactive compound derived from grapefruits, has been found to exhibit significant antineoplastic effects by inhibiting growth of various tumor cells including those of multiple myeloma, prostate, breast, hepatocellular carcinoma and neuroblastoma [26,27], but its effect on the EMT process in lung cancer cells is not well characterized.

We report here that BGM can significantly inhibit TGF-β-induced EMT and invasive capability of lung cancer cells, and the mechanism underlying these effects can correlate to its ability to abrogate the activation of PI3K/Akt/mTOR kinases. The inhibitory effects of BGM were first analyzed on the alterations in the expression level of EMT-related markers. Our results showed that BGM downregulated the levels of pro-EMT markers while upregulating those of anti-EMT markers in lung cancer cells without exhibiting any adverse effects.

During the EMT process, the expression of key tight-junction transmembrane protein, occludin, and junctional adhesion molecule, E-cadherin, has been reported to be downregulated. Loss of E-cadherin is considered as an important hallmark of EMT [42] and downregulation of occludin has been shown to be closely linked with the metastasis of tumor cells and has been found to be associated with EMT progression [43,44]. Interestingly, the repression of E-cadherin by snail or twist has also emerged as one of the critical steps driving the EMT process [45]. For example, snail can effectively bind to E-cadherin promoter, which in turn can repress its transcription [46]. Twist has also been found to be another important regulator of EMT in cellular as well as preclinical models of metastatic carcinomas [47]. Twist can also cause transcriptional downmodulation of the E-cadherin protein, and can cause an increase in the expression of diverse mesenchymal proteins [47].

We also examined the possible inhibitory effect of BGM on the TGF-β-induced EMT in lung cancer cells. TGF-β, an important cytokine, can also effectively regulate the EMT process [48]. During EMT, cells undergo a series of morphological changes that can lead to loss of epithelial features, and augmentation of the mesenchymal features subsequently causes induction of metastasis [30]. We found that the TGF-β-treated cells display morphological alterations from epithelial to mesenchymal phenotype in association with downregulation of occludin and E-cadherin levels that was concomitant with an increase of anti-EMT protein levels. BGM prevented morphological and molecular changes in TGF-β-stimulated cells. Indeed, our results indicate that BGM can significantly abrogate TGF-β-induced invasive capability in lung cancer cells.

Interestingly, activation of the PI3K/Akt signaling pathway is emerging as a central characteristic of the cells exhibiting the EMT process [49,50]. We observed that BGM could effectively downregulate the phosphorylation of various oncogenic kinases in lung cancer cells. We also analyzed the potential inhibitory effect of BGM on the TGF-β-stimulated phosphorylation of the PI3K/Akt/mTOR pathways, and data presented clearly indicated that BGM can substantially downregulate inducible activation of these kinases. A schematic diagram of how BGM targets these oncogenic kinases to abrogate the EMT process is depicted in Figure 4C.

In conclusion, this report highlights the inhibitory effects of BGM on the EMT process that is characterized by upregulation of adherence junction proteins and downregulation of mesenchymal markers as well as transcriptional repressors. Furthermore, BGM could also effectively suppress the metastasis through inhibiting the TGF-β-mediated EMT process by blocking phosphorylation of PI3K/Akt/mTOR kinases. Further studies are required to evaluate the potential effect of lower doses of BGM and validate its antimetastatic effects in appropriate preclinical models.
